# Genome-wide divergence, haplotype distribution and population demographic histories for *Gossypium hirsutum* and *Gossypium barbadense* as revealed by genome-anchored SNPs

**DOI:** 10.1038/srep41285

**Published:** 2017-01-27

**Authors:** Umesh K. Reddy, Padma Nimmakayala, Venkata Lakshmi Abburi, C. V. C. M. Reddy, Thangasamy Saminathan, Richard G. Percy, John Z. Yu, James Frelichowski, Joshua A. Udall, Justin T. Page, Dong Zhang, Tariq Shehzad, Andrew H. Paterson

**Affiliations:** 1 Gus R. Douglass Institute, Department of Biology, West Virginia State University, Institute, WV 25112-1000, USA; 2 USDA–ARS, Southern Plains Agricultural Research Center, 2881 F&B Road, College Station, TX 77845, USA; 3 WIDB, Plant and Wildlife Science Department, Brigham Young University, Provo, UT 84602, USA; 4 Plant Genome Mapping Laboratory, University of Georgia, 111 Riverbend Road, Room 228, Athens, GA 30605, USA

## Abstract

Use of 10,129 singleton SNPs of known genomic location in tetraploid cotton provided unique opportunities to characterize genome-wide diversity among 440 *Gossypium hirsutum* and 219 *G. barbadense* cultivars and landrace accessions of widespread origin. Using the SNPs distributed genome-wide, we examined genetic diversity, haplotype distribution and linkage disequilibrium patterns in the *G. hirsutum* and *G. barbadense* genomes to clarify population demographic history. Diversity and identity-by-state analyses have revealed little sharing of alleles between the two cultivated allotetraploid genomes, with a few exceptions that indicated sporadic gene flow. We found a high number of new alleles, representing increased nucleotide diversity, on chromosomes 1 and 2 in cultivated *G. hirsutum* as compared with low nucleotide diversity on these chromosomes in landrace *G. hirsutum*. In contrast, *G. barbadense* chromosomes showed negative Tajima’s D on several chromosomes for both cultivated and landrace types, which indicate that speciation of *G. barbadense* itself, might have occurred with relatively narrow genetic diversity. The presence of conserved linkage disequilibrium (LD) blocks and haplotypes between *G. hirsutum* and *G. barbadense* provides strong evidence for comparable patterns of evolution in their domestication processes. Our study illustrates the potential use of population genetic techniques to identify genomic regions for domestication.

One of the most remarkable stories in crop domestication is the origin of cultivated cotton^1^. *Gossypium* is remarkably diverse, with more than 50 species classified into 8 diploid genome groups and a single, monophyletic tetraploid cluster^2^,^3^,^4^,^5^,^6^. For many years, allopolyploid cotton has been used for evolutionary investigations into the genomic mysteries of polyploidy^2^. Recent phylogeny studies further resolved the monophyletic cluster of tetraploids consisting of six different species; *Gossypium mustelinum* (AD4) is the basal clade of allotetraploid taxa, all of which are direct descendants of an allopolyploidization event involving ancestral diploid species closely resembling *G. arboreum* (A2 genome diploid) and *G. raimondii* (D5 genome diploid) *G. tomentosum* (AD3), along with two other close species G. *ekmanianum* (AD6) and *G. hirsutum* (AD1), form a second clade that is sister to a third clade composed of the Galapagos Islands endemic *G. darwinii* (AD5) and *G. barbadense* (AD2)^7^.

Cultivars of *G. hirsutum* and *G. barbadense* produce the overwhelming majority of the world’s cotton fiber and oil. *G. hirsutum* has a large indigenous range encompassing most of Mesoamerica and the Caribbean, where it shows a diverse array of morphological forms spanning the wild-to-domesticated continuum^5^,^8^,^9^,^10^. Modern, improved varieties of *G. hirsutum* (“Upland cotton”) account for about 90% of world cotton commerce, and are day-length neutral annuals derived from subtropical, perennial photoperiodic landraces^9^,^11^. Wendel, *et al*.^9^ identified two centers of diversity for *G. hirsutum*, one in southern Mexico–Guatemala and the other in the Caribbean. *G. barbadense* cultivars are valued for their production of high quality fiber, classified as extra-long staple (ELS), and characterized by being exceptionally long, strong, and fine. These cottons are denoted variously as Pima, Egyptian or Sea Island cotton. *G. barbadense*, like *G. hirsutum*, spans a wild-to-domesticated range, and originated as a tropical, photoperiodic (short-day flowering) perennial. Originating in Peru and Ecuador, *G. barbadense* was domesticated in northwest South America and the Caribbean^1^,^12^,^13^,^14^.

Groupings and categorizations have been made within the wild to landrace continuum of both *G. hirsutum* and *G. barbadense* based upon phenotype and geographic origin. In *G. hirsutum*, races *yucatanense* and *punctatum* are two adjacent populations located in the inferred domestication region, the Yucatan peninsula in Mesoamerica^5^. Several localized derivatives of race *punctatum* include *richmondi, morilli, palmeri, marie*-*galante* and *latifolium*^15^,^16^, hereafter referred to as *G. hirsutum* landraces. These landraces have not differentiated at the molecular level. *G. barbadense* most likely underwent a trans-Andean expansion into northern South America after primary domestication west of the Andes^17^,^18^. A secondary stage of dispersal involved expansion into Central America, the Caribbean and the Pacific^18^. During this dispersal, enough accumulated differentiation occurred within the species to produce the sibling Galapagos Islands endemic species *G. darwinii*, and the distinctive variety *braziliense*, formerly recognized as the species *G. braziliense*^13^.

The molecular diversity of both *G. hirsutum* and *G. barbadense* has been extensively studied with a wide variety of marker tools^11^,^19^,^20^,^21^,^22^,^23^,^24^,^25^,^26^,^27^,^28^,^29^,^30^,^31^. However, with the recent release of two independent whole-genome sequences from the same genotype, TM-1, a highly homozygous line of allotetraploid *G. hirsutum*^32^,^33^, researchers have rich opportunities to explore genome-wide constraints on molecular diversity and unravel domestication mechanisms of tetraploid cultivated and landrace cotton.

This report explores at a new level of resolution how breeding histories and selective pressures shaped the gene pools of *G. hirsutum* and *G. barbadense* cotton. We used genotyping-by-sequencing (GBS) to generate 10,129 single nucleotide polymorphisms (SNPs) for 440 *G. hirsutum* and 219 *G. barbadense* accessions from representative samples of cultivated and wild pools. This report focuses on a comparative study of linkage disequilibrium (LD) among *G. hirsutum* and *G. barbadense* chromosomes and constraints in population structure of both allopolyploids. With this genome-wide characterization of SNP variation in domesticated tetraploid cotton, we show that cultivated allotetraploid species experienced both unique and common patterns of genome-wide selection pressure during domestication.

## Materials and Methods

### Germplasm

A representative sample of 658 cotton accessions (440 of *G. hirsutum* and 218 of *G. barbadense*) collected from 85 countries in North America, South America, Europe, Asia, and Africa were obtained from the National Cotton Germplasm Collection (NCGC) maintained by the USDA-ARS in College Station TX^34^ (Table S1). Improved and unimproved *G. barbadense* (86 and 132 accessions, respectively) and *G. hirsutum* (268 and 172 accessions, respectively) were selected for inclusion. Selection of accessions was performed with great attention to achieving a representative sample of the geographic, developmental and historic diversity present in the collection.

### Genotyping by sequencing

Genomic DNA isolation from the seedlings involved the DNeasy plant mini kit (QIAGEN, Germany), and GBS was as described^35^,^36^. DNA was treated with the restriction enzyme *Pst*1, barcoded by accession, and sequenced on an Illumina HiSeq 2000 as described^35^. Raw sequence reads were trimmed with use of Sickle (https://github.com/najoshi/sickle), with minimum PHRED quality threshold of 20 in a sliding window. Then reads were demultiplexed and barcodes were removed. Trimmed reads were mapped to the *G. raimondii* and *G. hirsutum* reference sequences by using GSNAP with SNP-tolerant mapping based on >20 million homoeo-SNPs^37^. Aligned reads were processed by use of SAMtools and classified as the *G. hirsutum* A_T_- and D_T_-genomes by use of PolyCat^38^,^39^. Barcoded sequence reads were processed and collapsed into a set of unique sequence tags, with one TagCounts file produced per input FASTQ file^40^. The separate TagCounts files were then merged to form a “master” TagCounts file, which retained only those tags present at or above an experiment-wide minimum count. This master tag list was then aligned to the TM-1 (*G. hirsutum*) reference genome^33^ and a Tags On Physical Map (TOPM) file was generated, containing the genomic position of each tag with a unique, best alignment. The barcode information in the original FASTQ files was then used to tally the number of times each tag in the master tag list was observed in each sample (“taxon”) and these counts were stored in a TagsByTaxa (TBT) file. The information recorded in the TOPM and TBT was then used to discover SNPs at each “TagLocus” (set of tags with the same genomic position) and filter the SNPs based upon the proportion of taxa covered by the TagLocus and minor allele frequency^40^. For each retained SNP, the allele represented by each tag in the corresponding TagLocus was recorded in the TOPM file, along with its relative position in the locus. The end product of the Discovery Pipeline was a “production-ready” TOPM that was then used by the production Pipeline to call SNPs. To call SNPs and ensure that indels were handled consistently, a *de novo* multiple sequence alignment of all the tags in each TagLocus was performed using the BioJava 3.0 API^41^, which implements the CLUSTAL W algorithm^42^. For each SNP in the resulting “TagLocusAlignment”, the allele represented by each tag is determined and the TBT file was consulted to tally the observed depths of each allele in each taxon.

### Population structure analysis

Genetic diversity values were calculated by a neighbor-joining algorithm with TASSEL 5. In a second approach, we used identity by State (IBS) and principle component analysis (PCA) with the SNP & Variation Suite (SVS v8.1.5) (Golden Helix, Inc., Bozeman, MT, USA; www.goldenhelix.com). Observed nucleotide diversity (π) and Tajima’s D was estimated by using TASSEL v5.0 with a sliding-window approach as described^43^. Estimation of the fixation index (*F*_*ST*_) were based on the Wright F statistic^44^ calculated by use of SVS v8.1.5.

### Characterization of LD

We considered only SNPs mapped to the *G. hirsutum* whole-genome sequence draft, because knowing the chromosome location of SNPs helps prevent spurious LD and characterization of sub-genomic LD. Haplotype blocks were calculated for all markers by using the default settings in SVS v8.1.5 as described in Nimmakayala, *et al*.^45^. Adjacent and pairwise measurements of LD were calculated separately for each chromosome. For computing linkage disequilibrium (LD), we used expectation-maximization (EM) algorithm, formalized by^46^, is a iterative technique for obtaining maximum likelihood estimates of sample haplotype frequencies.

## Results and Discussion

### GBS of tetraploid cotton and SNP mining

A total of 1,700,626,447 reads were available for analysis after quality trimming; 471,553,537 (33.2%) were classified as from the A_T_-genome and 460,910,405 (32.4%) from the D_T_-genome (Fig. S1). The remainder could not be classified by known SNPs between genomes (homoeo-SNPs). The median value of reads per genome (A_T_ and D_T_) for each accession was 519,563.5; 2,240,812 (46.0%) were aligned to unique positions and 1,020,968 (20.9%) to multiple positions. Overall, 1,612,880 (33.1%) could not be aligned to the *G. hirsutum* reference genome. When orthologs were below a certain threshold of divergence, sequences aligned to both A and D genomes, which led to multiple-site–aligned sequence tags. In this case, sequence tags from orthologous sequences would collapse into a single locus or two separate loci. The SNPs from these loci showed very high heterozygosity, depending on the sequencing depth. Figure S2 shows the distribution of heterozygosity (in individuals and SNPs) for the SNPs for the *G. raimondii* and *G. hirsutum* genomes. The distribution of heterozygosity for the SNPs, when mapped to the *G. raimondii* genome was higher. In total, 10,129 SNPs were called for uniquely aligned sequence tags and hence are singletons: 314, 240, 327, 205, 641, 247, 315, 448, 326, 441, 567, 454, 367, 309, 364, 203, 317, 643, 296, 326, 451, 422, 434, 548, 485 and 369 were mapped to Chrs. 1–26, respectively. We found SNPs at average intervals of 195 kb across the *G. hirsutum* genome.

### Population structure, nucleotide diversity and demographic history

**Figure 1:**
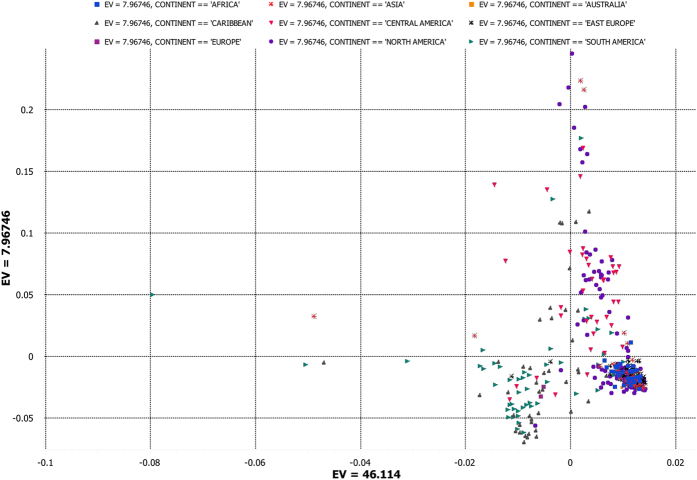
First and second components of principal component analysis (PCA) of 2,084 single nucleotide polymorphisms (SNPs) within a set of global *G. hirsutum* accessions (SA: 268 Upland cultivars; TX: 172 landraces). See Table S3 for a list of accessions and respective eigen values for respective positions of individual accessions in the figure.

To investigate genetic differentiation due to population structure among cotton groups as reflected by these genome-wide SNPs, we used PCA and NJ analysis. In a PCA of all analyzed accessions, most cultivated *G. hirsutum* lines clustered together and 172 photoperiodic landraces of *G. hirsutum* (TX) clustered into two groups. One group of TX lines was predominantly from South America and the second group was from Central America and the Caribbean (Fig. 1 and Table S3). The cluster of Upland cotton cultivars spread into the cluster of Central America and Caribbean landraces, which indicated that this region might be the center of domestication for Upland cotton. In PCA of 268 Upland cotton cultivars representing worldwide breeding efforts, the distribution of U.S. cultivars spanned the collective distributions of all other national cultivars, indicating a level of diversity equal to the global cultivar diversity. An additional PCA of only 219 *G. barbadense* lines clearly separated cultivars and landraces (Fig. S3 and Table S4).

**Figure 2:**
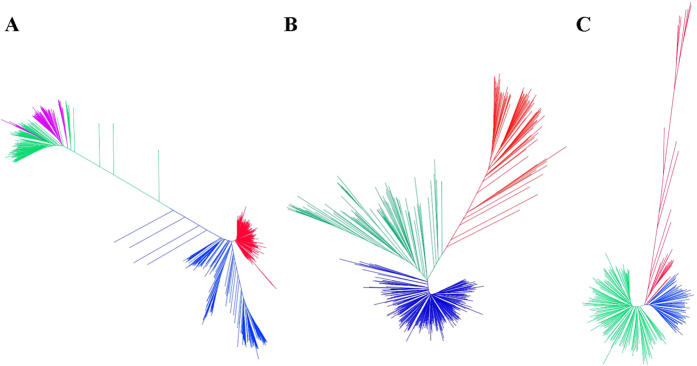
Phylogenetic trees constructed with neighbor-joining for (**A**) both *Gossypium hirsutum* cultivars (red: Upland cultivars; blue: landraces) and *Gossypium barbadense* cultivars (magenta: cultivated; green: landraces) and (**B**) cultivars and wild *G. hirsutum* (green: Upland cultivars; blue and red: two clusters of landraces) and (**C**) cultivars and landraces of *G. barbadense* (blue: cultivated; green and red: two clusters of landraces). Distance matrix is in Table S2.

PCA (Fig. 1) and NJ analysis (Fig. 2A, Table S2) revealed two distinct clusters for the *G. hirsutum* and *G. barbadense* species, with a clear separation of cultivated and landraces within each species. A focused NJ analysis of only *G. hirsutum* (Fig. 2B) revealed three distinct clusters within the species, with one cluster comprised of cultivated Upland cottons, an adjacent cluster of landrace accessions from Central America and the Caribbean, and a third cluster of predominantly South American accessions. Overall genetic diversity among the allotetraploids was 0.336, with mean genetic diversity 0.089, 0.22, 0.173 and 0.336 among cultivated *G. hirsutum*, landraces of *G. hirsutum*, cultivated *G. barbadense*, and landraces of *G. barbadense*, respectively. The diversity among cultivated *G. hirsutum* accessions is more or less evenly distributed across the world with the highest mean diversity estimates of 0.098, 0.08, 0.071, 0.081, 0.078 and 0.072 occurring among accessions from South America, Central America, the United States, Europe, Asia and Africa, respectively (Fig. 2B). A focused NJ analysis of only *G. barbadense* (n = 219) lines resolved a monophyletic cultivar cluster containing 86 accessions of cultivated *G. barbadense* (Fig. 2C) and placed the landraces of *G. barbadense* into two distinct clusters. The cluster in close proximity to the cultivated forms was a mixture of landraces from the Caribbean and South America. This analysis of population genomic diversity in cultivated allotetraploid genomes of cotton adds resolution to prior knowledge with the inclusion of more accessions and a large genomewide sampling of SNPs. Some studies have developed high-throughput SNPs for tetraploid cultivated cotton; most focused on generating high-resolution genetic maps^36^,^37^,^38^,^39^,^40^,^41^,^42^,^43^. Our study involved the use of GBS-generated SNPs mapped to the recently released *G. hirsutum* genome sequence^33^ to identify singleton SNPs assigned to chromosome positions.

We examined allele sharing across the panel by calculating identity-by-state (IBS) coefficients among all pairs of accessions^47^. The mean IBS sharing within cultivated and landrace *G. hirsutum* was 0.95 and 0.85, respectively. The lowest IBS sharing within the cultivated *G. hirsutum* combinations was noted for Paymaster 54 and Hopi (0.62), Zhong Mian Suo Hao and Hsing-Tai #68–71 (0.63), Express 432 and Hopi (0.63); Rilcot and Hopi (0.63); Rowden #2 and Kekchi (0.63), Blight Master and Anton Stormproof 99 and Lankart Sel.611 (0.63), UKA B1- (72) 047 and UK 64 (0.63), UKA S1 (72) 070 and Deltapine 16 (0.63), UA 7–10 and Hopi (0.63), and 108F with several other *G. hirsutum* accessions (0.63). The low IBS values obtained between a number of cultivars and Hopi and Kekchi are unsurprising, in that Hopi and Kekchi cottons are probably most appropriately classified as primitive cultivars. Hopi cultivars result from pre-Columbian cultivation by the Hopi Indians of cotton obtained from Mexico, and Kekchi cotton was imported into the U.S. from Mexico in the early 20^th^ century in conjunction with efforts to find boll weevil resistance. These relatively modern introductions to cultivation have contributed to a wider divergence in cultivated cotton. IBS sharing suggests a high degree of genetic similarity between the two accessions/species, either intentionally or unintentionally. The mean IBS sharing between cultivated and landrace *G. hirsutum* was 0.55. Average IBS sharing between cultivated *G. hirsutum* and cultivated *G. barbadense* was 0.41. *G. hirsutum* cultivated genotypes 108 F and M100 showed high IBS sharing, from 0.92 to 0.95, with Old Pima, Yuma, Bolivia, Pima (Raleigh Stock), Linia1780, Tadla116, Pima32, Pima S-7, 8810, Pima S-3, Early Pima, P76 and Pima S-1, among *G. barbadense* cultivars. Perhaps some crossing between tetraploid species occurred to produce the 108 F and M100 genotypes because of their high IBS value and historical efforts of trait introgression. Given the high IBS value, an equally probable scenario may be that 108 F and M100 are truly *G. barbadense* and have been misclassified as *G. hirsutum*. The mean observed IBS sharing within cultivated *G. barbadense* was 0.89. *G. barbadense* cultivars Bahamas 1 and St. Croix consistently showed low IBS sharing (0.53 to 0.58) with the remaining *G. barbadense* accessions, and may be indicative of low introgression into these two accessions, or their misclassification.

Our diversity and IBS analyses of the two cultivated allotetraploids showed little sharing of alleles between the genomes, with only a few combinations showing high IBS sharing, which indicates sporadic introgression between the two species. Because the two allotetraploids were domesticated independently in geographically isolated locations, each thousands of years ago^6^,^15^,^48^, such divergence is expected.

**Figure 3:**
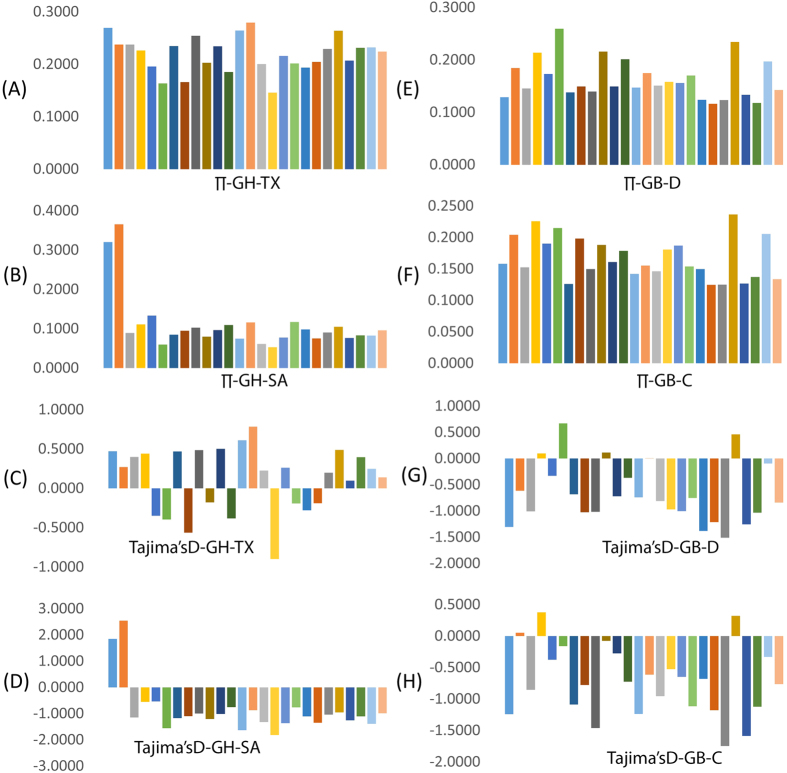
Frequency spectrum for the chromosomal means of nucleotide diversity (π) and Tajima’s D for cultivated and non-cultivated *G. hirsutum* and cultivated and non-cultivated *G. barbadense*.

Because of the strong population structure, we assessed patterns of variation separately for each group when making inferences about the evolutionary dynamics of domestication. Crop domestication is often associated with ‘population bottlenecks’, due to the limited number of founding individuals experiencing domestication events. These bottlenecks may be evident in cotton when comparing diversity between elite cultivars and wild or minimally improved landraces. The within-group polymorphic SNP number was 2,084 (minor allele frequency [MAF] ≥ 0.05) for *G. hirsutum*, and 2417 (MAF ≥ 0.05) for *G. barbadense*. To assess levels and patterns of polymorphism in the cultivated (SA) and landrace (TX) *G. hirsutum* and cultivated (C) and wild (D) *G. barbadense* gene pools, we estimated nucleotide diversity (π) and Tajima’s D across various chromosomes using the SNPs that have MAF of ≥0.05. The frequency of segregating SNPs as reflected by various chromosomal measures of mean nucleotide diversity and Tajima’s D is presented in Fig. 3. We found a high number of new alleles, representing nucleotide diversity, on chromosomes 1 and 2 in cultivated *G. hirsutum* as compared with low nucleotide diversity on these chromosomes in landrace *G. hirsutum*. Subsequent spread of *G. hirsutum* cultivars across the world and exposure to diverse breeding programs or selection might explain such rapid population size expansion due to overrepresentation of new alleles segregating in cultivars. In addition, we noted biased distribution of Tajima’s D toward negative values on the remaining chromosomes (3 to 26) in cultivated *G. hirsutum* as compared with its wild ancestor, which indicates a population bottleneck during domestication. In contrast, *G. barbadense* chromosomes showed negative Tajima’s D on several chromosomes for both cultivated and landrace types, which indicate that speciation of *G. barbadense* itself might have occurred with relatively narrow genetic diversity. For cultivated *G. barbadense*, chromosome 4 and 22 appears to have new alleles for nucleotide diversity and Tajima’s D was more positive than for unimproved types.

The presence of high diversity on chromosomes 1 and 2 in cultivated *G. hirsutum* indicates many relatively new alleles in this region. In contrast, for cultivated *G. barbadense*, chromosome 4 appears to show increased nucleotide diversity and Tajima’s D. Unusually divergent genomic regions resulting from disproportionate accumulation of novel alleles on particular chromosomes among closely related sub-species such as *indica* and *japonica* in rice, *G. hirsutum* and *G. barbadense* cottons and *Capsicum annuum* and *C. baccatum* peppers could cause reproductive barriers or incompatibility resulting in partial fertility^45^,^49^. Similar to current findings, several reports implicated newly recruited polymorphisms as causing highly divergent genomic regions that may control traits associated with reproductive incompatibility or ecological adaptation^45^,^50^,^51^.

**Figure 4:**
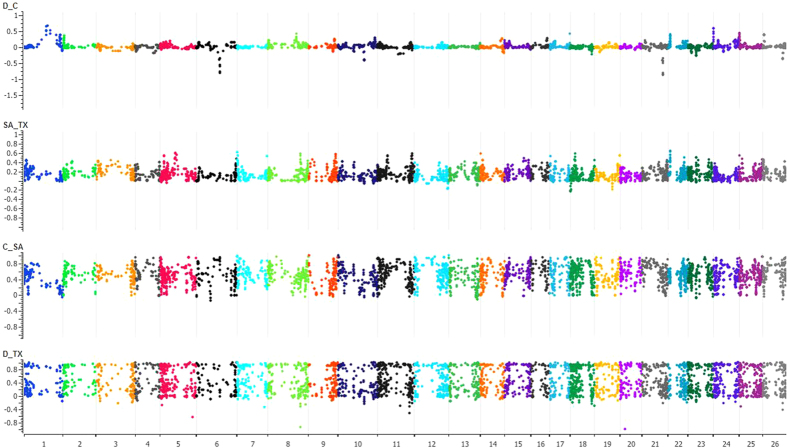
Manhattan plots of chromosome-wise pairwise *F*_*ST*_ values (mean smoothed) for D_C: (*G. barbadense* landraces and *G. barbadense* cultivated); SA_TX (*G. hirsutum* Upland cultivars and uncultivated *G. hirsutum*); C_SA (cultivated *G. barbadense* and cultivated *G. hirsutum*) and D_TX (landraces of *G. barbadense* and landraces of *G. hirsutum*).

To further resolve species differentiation between the two tetraploid taxa, we estimated pairwise fixation index (*F*_*ST*_) across all polymorphisms with MAF ≥ 0.05. All *F*_*STs*_ were highly significant (*P* > 0.001). The *F*_*ST*_ between wild accessions of *G. hirsutum* and *G. barbadense* was 0.66 and 0.53, respectively. The *F*_*ST*_ between cultivated and wild accessions within *G. hirsutum* was 0.20 and between cultivated and landraces within *G. barbadense* was 0.05. Genome-wide means smoothed *F*_*ST*_ values for various groups of taxa as presented for all chromosomes in Manhattan plots, showing genomic regions with spikes representing the highest *F*_*ST*_ values and sweeps the lowest (Fig. 4). In recent studies, because of potential *F*_*ST*_ outliers, adaptive divergence is assessed by comparing neutral genetic variation as outlined by Lewontin-Krakauer method^52^ with quantitative genetic differentiation (*Q*_*ST*_) to infer the cause for population differentiation^53^,^54^. Whitlock (2008)^55^ has emphasized that *Q*_*ST*_ values should be compared to the distribution of *F*_*ST*_ values as Lewontin-Krakauer method by itself does not work well for *F*_*ST*_ values above 0.1 such as in the current study. We have currently undertaken GWAS in multiple locations and years for fiber components in *G. hirsutum* and *G. barbadense* for elucidating evolutionary potential of various genomic regions under divergent selection.

### Haplotypes, LD decay, and comparative analysis of species-specific versus common LD blocks

**Figure 5:**
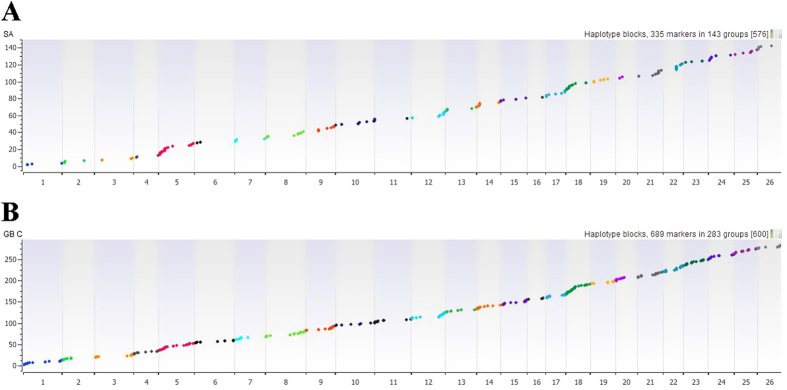
Haplotype distribution across various chromosomes (**A**) within cultivars of *G. hirsutum* and (**B**) within *G. barbadense* cultivar accessions. Detailed haplotype tables for *G. hirsutum* and *G. barbadense* are in Tables S7 and S8, respectively.

Haplotype distribution is important in comparing common and unique patterns of genetic variation of allotetraploid cotton gene pools and has a wide range of applications. Domestication process and breeding history are the two major processes that shape haplotype structure^45^. We used “Minimize historical recombination”, a block-defining algorithm developed by Gabriel, *et al*.^56^. The upper and lower confidence bound were set to 0.98 and 0.70, respectively, and SNPs below MAF of 0.05 were skipped. Maximum block length was set to 160 Kb. The EM (Expectation Maximization) algorithm was used for haplotype estimation with convergence tolerance 0.0001 and frequency threshold of 0.01. Maximum EM iterations were set to 50. We identified 335 SNPs in 143 haplotypes within cultivated *G. hirsutum* and 689 SNPs in 283 haplotypes (Table S7) in cultivated *G. barbadense* (Table S8). The location of haplotypes was well conserved in both species, whereas the length of haplotypes was longer for *G. barbadense* than *G. hirsutum*, which may indicate narrow genetic diversity in *G. barbadense* (Fig. 5).

**Figure 6:**
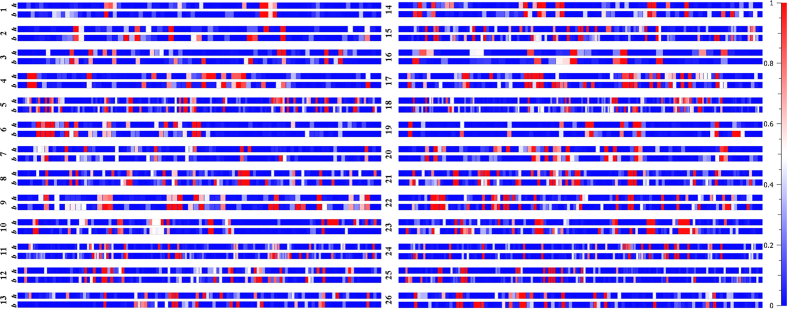
Comparative analysis of LD block distribution across various chromosomes in *G. hirsutum (h*) and *G. barbadense (b*). Heat map depicts strength of association.

Conserved genomic positions of haplotype formation in both species may indicate conserved patterns of evolution. Correspondence of occurrence of shared haplotype blocks was tested using the hypergeometric probability function (*P* = 6.29E-26). We estimated LD by using an entire marker set with MAF ≥ 0.05 and identified 1419 and 1293 significant associations in *G. hirsutum* and *G. barbadense* respectively (Tables S5 and S6). We also estimated chromosome-wise distribution of LD blocks separately for *G. hirsutum* and *G. barbadense* (Figs 6 and S4). Although the magnitude and strength of association differed among the common LD blocks between the cultivated cotton genomes, most of the blocks appeared to be coincident. LD decay in *G. hirsutum* occurred over intervals averaging 117 Kb. In *G. barbadense*, 1,293 SNP associations suggested a genome-wide LD decay over intervals averaging 128 Kb (Table S6). Chromosome-wise shared LD blocks between *G. hirsutum* and *G. barbadense* and hypergeometric probability significance levels for correspondence between the species are presented in Table 1. The locations of singleton SNPs in the tetraploid genomes provided a unique opportunity to unravel several interesting findings apart from characterizing the global genome-wide diversity of *G. hirsutum* and *G. barbadense*. Using genome-wide SNPs, measurements of genetic diversity, haplotype distribution and LD decay patterns in the two genomes shed light on genomic architecture, domestication processes and population demographic history.

Estimates of LD across the 26 chromosomes of tetraploid cotton provide insights into the haplotype block structure of the various chromosomes, providing researchers with a way to efficiently select markers and infer genotypes based on nearby loci. When a new haplotype-containing accession is used in a breeding program, it can recombine with other diverse haplotypes to create progenies of intermediate relatedness, a genetic basis for novel variation^57^. Also of interest to breeders, our diversity and population structure analysis provides insights into the domestication process between *G. hirsutum* and *G. barbadense* useful for controlling error due to population stratification, which often leads to identifying spurious associations in GWAS experiments. Our analysis of LD revealed considerable similarity within and across the two genomes. In some instances, LD remained elevated over extended genomic regions in both species. In sunflower, Mandel, *et al*.^58^ noted that large LD blocks occurred in close proximity to genes and QTL for traits targeted by selection during domestication or improvement. Given that our analysis was based on only 3,708 SNPs, we probably missed several LD blocks. Future resequencing of several diverse cultivated accessions of *G. hirsutum* and *G. barbadense* would precisely characterize LD decay and distribution.

Our study illustrates the potential for breeders to use population genetic techniques to identify genomic regions of importance. Performing GWAS for fiber traits with dense SNP panels is feasible and would provide further insights into the complexity of genome organization underlying these two allotetraploid cotton species.

## Additional Information

**How to cite this article:** Reddy, U. K. *et al*. Genome-wide divergence, haplotype distribution and population demographic histories for *Gossypium hirsutum* and *Gossypium barbadense* as revealed by genome-anchored SNPs. *Sci. Rep.* **7**, 41285; doi: 10.1038/srep41285 (2017).

**Publisher's note:** Springer Nature remains neutral with regard to jurisdictional claims in published maps and institutional affiliations.

## Supplementary Material

Supplementary Information

Supplementary Figure S1

Supplementary Figure S2

Supplementary Figure S3

Supplementary Figure S4

Supporting Tables

## Figures and Tables

**Table 1:** Chromosome-wise shared LD blocks between *G. hirsutum* and *G. barbadense* and significance levels for correspondence.

Chromosome	Total LD Blocks	Shared*	Hypergeometric Probability
1	31	9	0.00458
2	27	12	0.00010
3	34	9	0.00320
4	32	12	0.00130
5	67	37	1.29E-07
6	37	16	0.00041
7	30	12	0.00021
8	47	20	5.87E-05
9	37	18	7.53E-08
10	38	18	2.58E-03
11	62	25	3.67E-08
12	48	25	0.00017
13	49	14	0.00343
14	42	13	0.00020
15	58	24	7.54E-07
16	19	6	0.11068
17	47	25	4.05E-05
18	67	27	2.40E-08
19	19	13	0.00386
20	40	16	8.45E-05
21	53	25	6.65E-07
22	50	24	3.43E-06
23	47	13	1.77E-05
24	48	24	3.88E-05
25	53	22	1.39E-07
26	42	14	0.00037

^*^Shared between *Gossypium barbadense* and *Gossypium hirsutum*.
