# Supplementary material for: Genome-wide divergence, haplotype distribution and population demographic histories for Gossypium hirsutum and Gossypium barbadense as revealed by genome-anchored SNPs
Source: Sci Rep. 2017 Jan 27;7:41285. doi: 10.1038/srep41285 (PMC5269598; doi:10.1038/srep41285)
Supplement: Supplementary Information [file srep41285-s1.pdf]

SUPPORTING INFORMATION:

Genome-wide divergence, haplotype distribution and population demographic histories for *Gossypium hirsutum* and *Gossypium barbadense* as revealed by genome-anchored SNPs

Umesh K. Reddy[1*†], Padma Nimmakayala[1], Venkata Lakshmi Abburi[1], C.V.C.M Reddy[1], Thangasamy Saminathan[1], Richard G. Percy[2†], John Z. Yu[2*], James Frelichowski[2], Joshua A. Udall[3], Justin T. Page[3], Dong Zhang[4], Tariq Shehzad[4], Andrew H Paterson[4†]

[1]Gus R. Douglass Institute, Department of Biology, West Virginia State University, Institute, WV 25112-1000, USA

[2]USDA–ARS, Southern Plains Agricultural Research Center, 2881 F&B Road, College Station, TX 77845, USA

[3]295 WIDB, Plant and Wildlife Science Department, Brigham Young University, Provo, UT 84602, USA

[4]Plant Genome Mapping Laboratory, University of Georgia, 111 Riverbend Road, Room 228, Athens, GA 30605, USA

***Contributed equally**

† Co corresponding authors

Umesh K. Reddy: 1-304-766-3066; ureddy@wvstateu.edu

Richard G. Percy: 1- 979-260-9310; Richard.Percy@ars.usda.gov

Andrew H. Paterson: 1-706-583-0162 paterson@uga.edu

Supporting figures

Figure S1: Coverage of SNPs in the A and D diploid genomes on mapping sequence reads to the diploid sequence drafts.

Figure S2: Differences in distribution of heterozygosity (in individuals and SNPs) when SNPs were mapped against whole genome sequences of *Gossypium hirsutum* and *Gossypium barbadense.*

Figure S3. Principal component analysis (PCA) of 2,417 single nucleotide polymorphisms (SNPs) among global *G. barbadense* accessions (C: cultivated; D: dooryard or landraces). See Tables S7 for the accessions and respective eigen values to locate individual accessions on the graphs.

Figure S4: Comparative analysis of linkage disequilibrium (LD) distribution across various chromosomes in *G. hirsutum* and *G. barbadense*. Heat map depicts strength of association.

Supporting tables

Table S1: List of *G. hirsutum* and *G. barbadense* cotton accessions used in the current study.

Table S2: Matrix of molecular diversity indices for 659 lines belonging to *G. hirsutum* and *G. barbadense*.

Table S3: Eigen values for the first three principle components estimated for various accessions belonging to *G. hirsutum*.

Table S4: Eigen values for the first three principle components estimated for various accessions belonging to *G. barbadense*.

Table S5: Linkage disequilibrium (LD) analysis for adjacent SNP pairs across the *G. hirsutum* genome.

Table S6: LD analysis for adjacent SNP pairs across the *G. barbadense* genome.

Table S7: Haplotype blocks across the cultivated *G. barbadense* genome.

Table S8: Haplotype blocks across the cultivated *G. hirsutum* genome..
