# Supplementary figures and images for: Genome-wide divergence, haplotype distribution and population demographic histories for Gossypium hirsutum and Gossypium barbadense as revealed by genome-anchored SNPs
Source: Sci Rep. 2017 Jan 27;7:41285. doi: 10.1038/srep41285 (PMC5269598; doi:10.1038/srep41285)

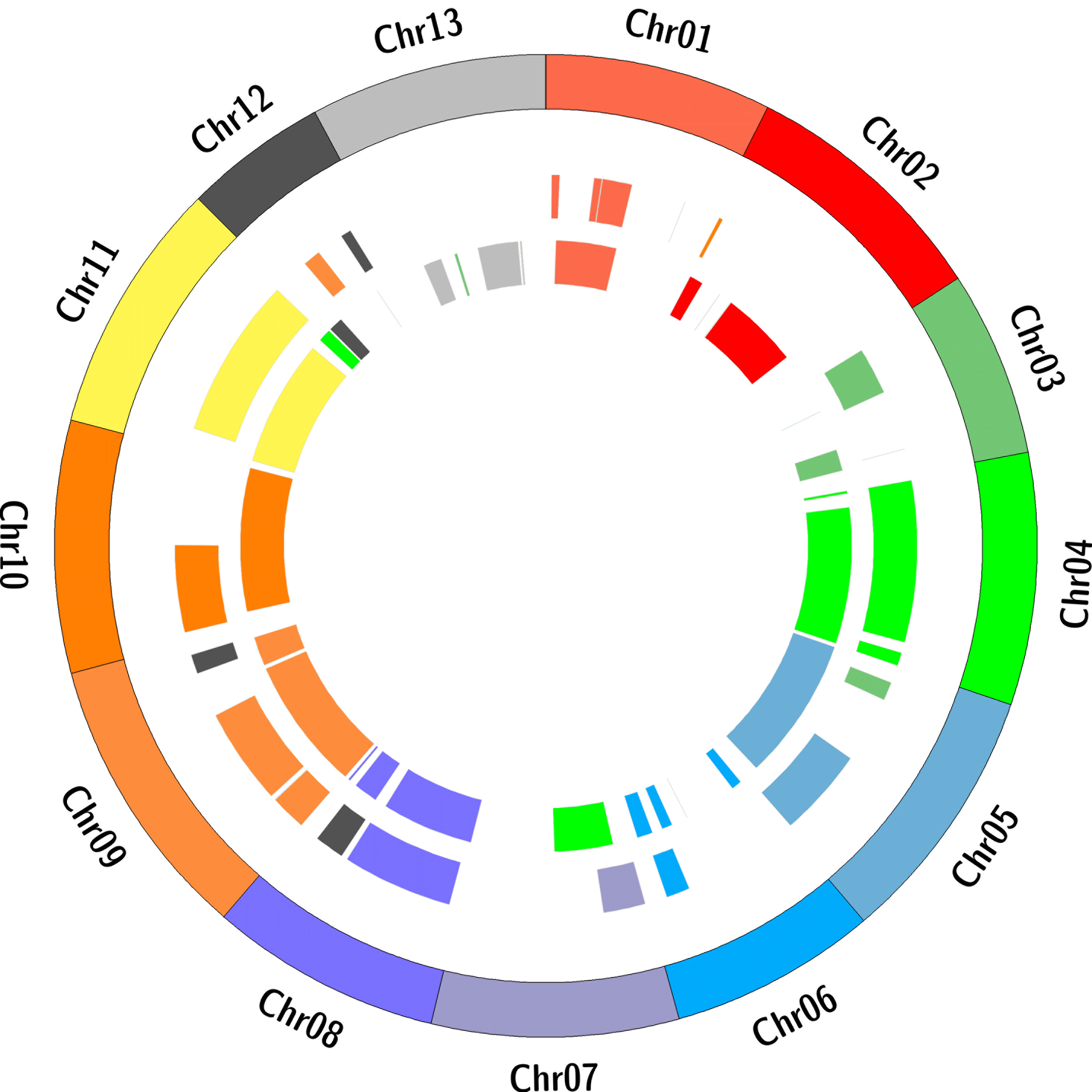

Supplement: Supplementary Figure S1 [file srep41285-s2.gif]

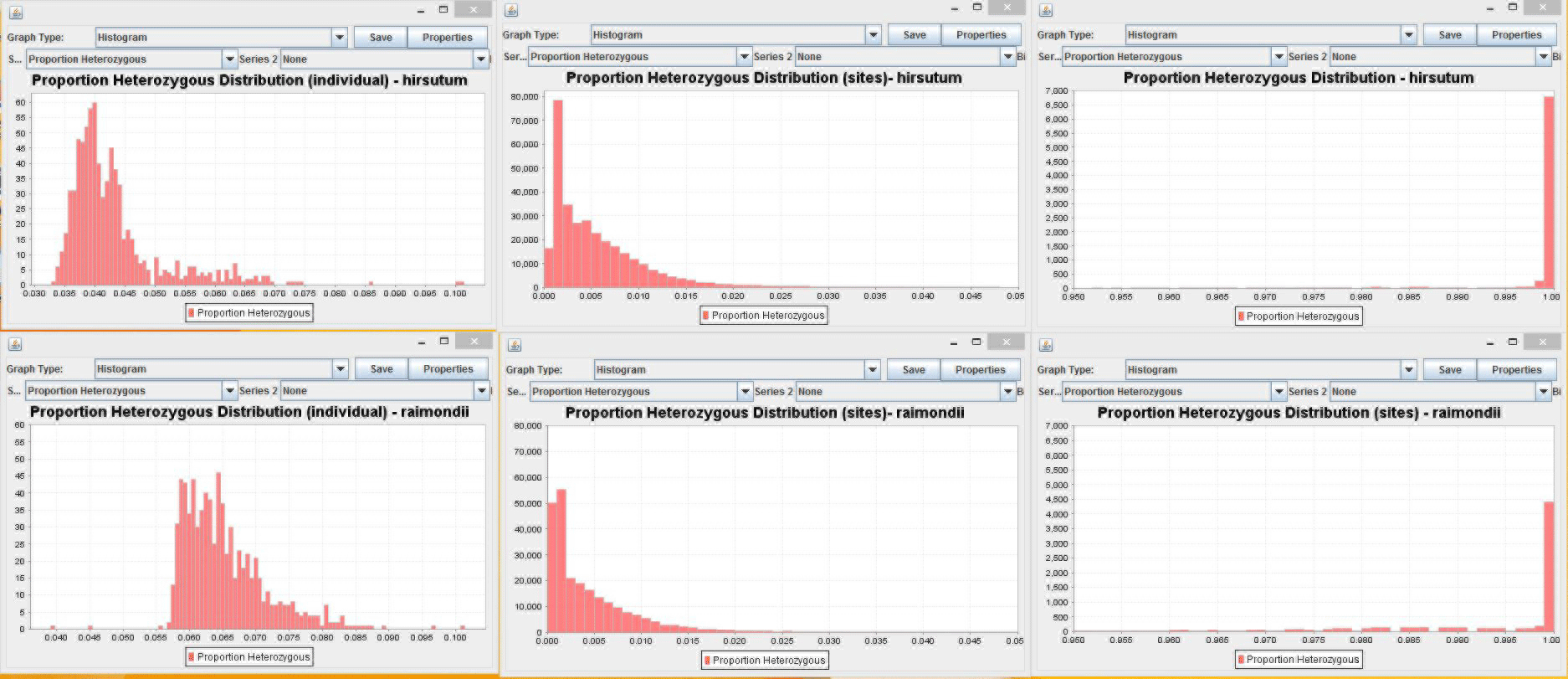

Supplement: Supplementary Figure S2 [file srep41285-s3.gif]

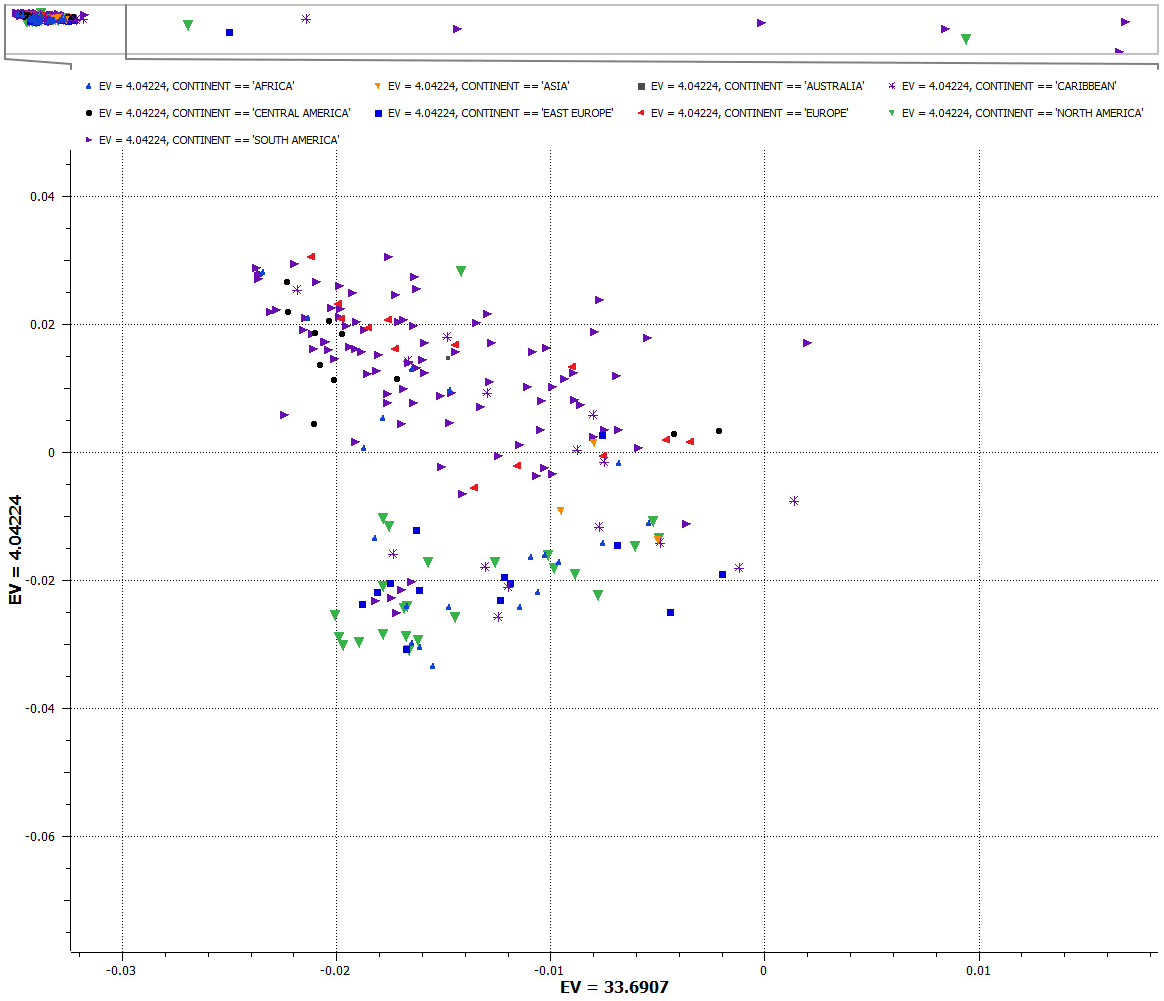

Supplement: Supplementary Figure S3 [file srep41285-s4.gif]
