# Supplementary material for: Genome-wide divergence, haplotype distribution and population demographic histories for Gossypium hirsutum and Gossypium barbadense as revealed by genome-anchored SNPs
Source: Sci Rep. 2017 Jan 27;7:41285. doi: 10.1038/srep41285 (PMC5269598; doi:10.1038/srep41285)
Supplement: Supplementary Figure S4 [file srep41285-s5.pdf]

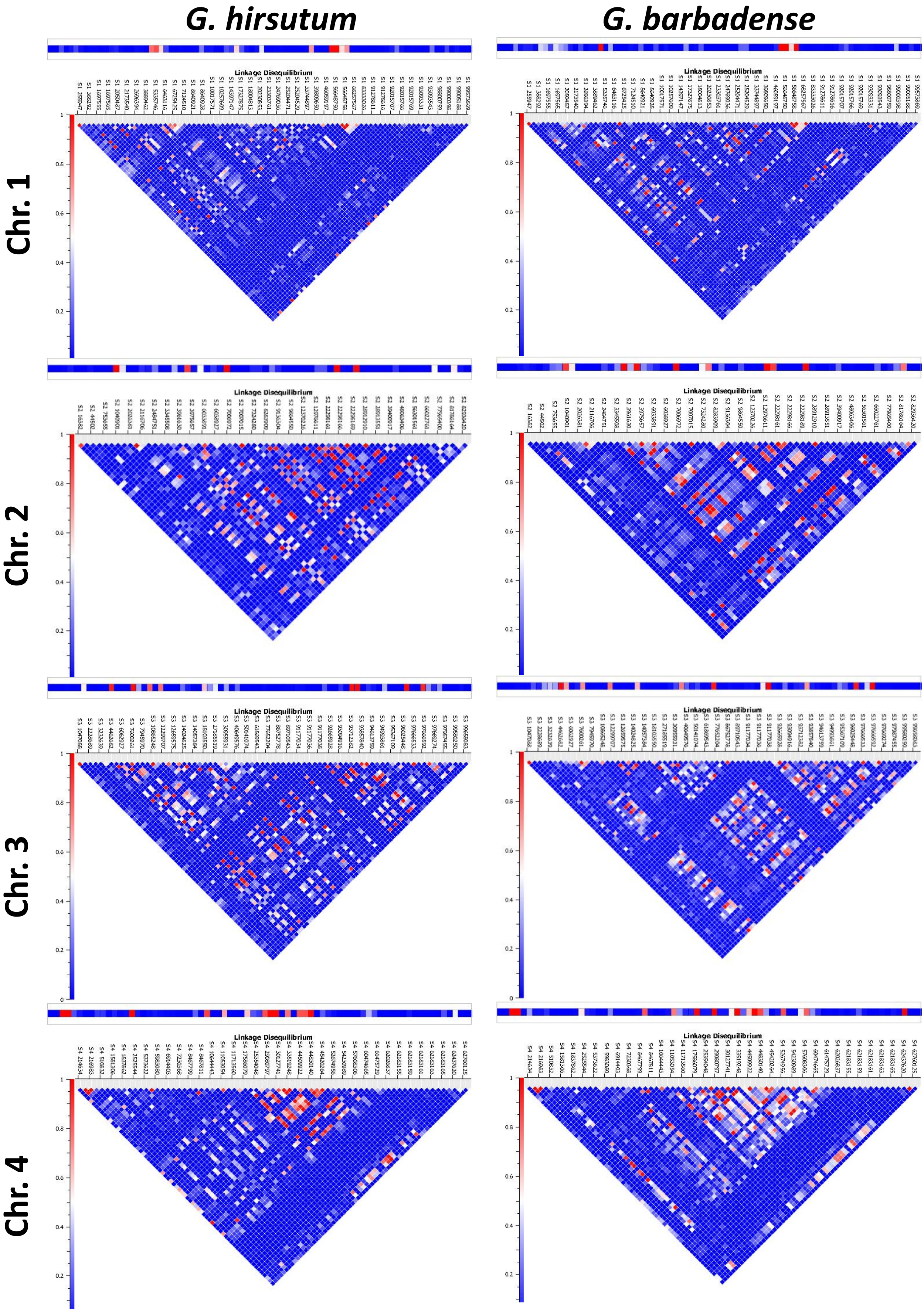
G. hirsutum
G. barbadense
Chr. 1
Chr. 2
Chr. 3
Chr. 4
Linkage Disequilibrium

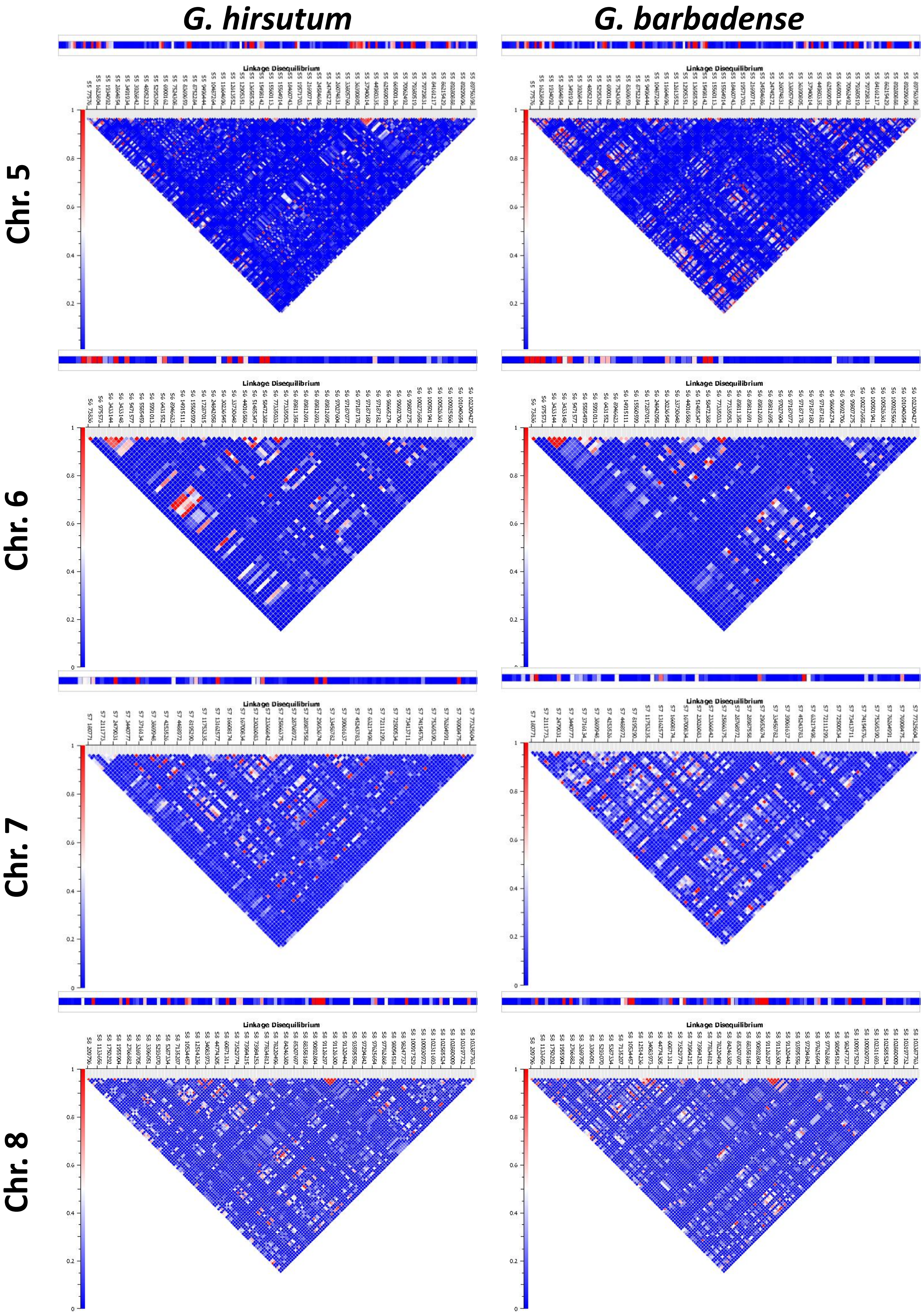

G. hirsutum
G. barbadense
Chr. 5
Chr. 6
Chr. 7
Chr. 8
Linkage Disequilibrium

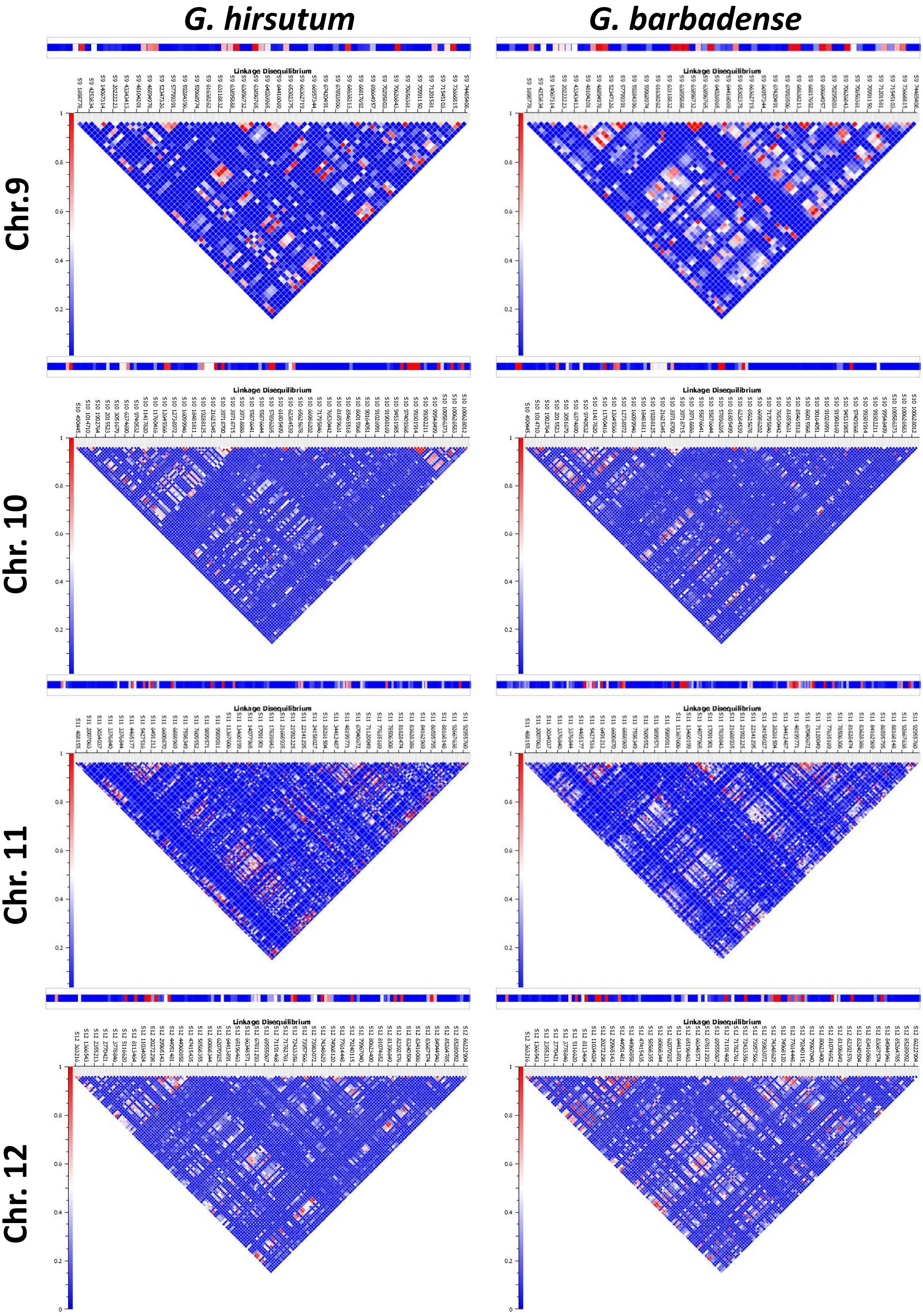
G. hirsutum
G. barbadense
Chr.9
Chr. 10
Chr. 11
Chr. 12
Linkage Disequilibrium
1
0.8
0.6
0.4
0.2
0

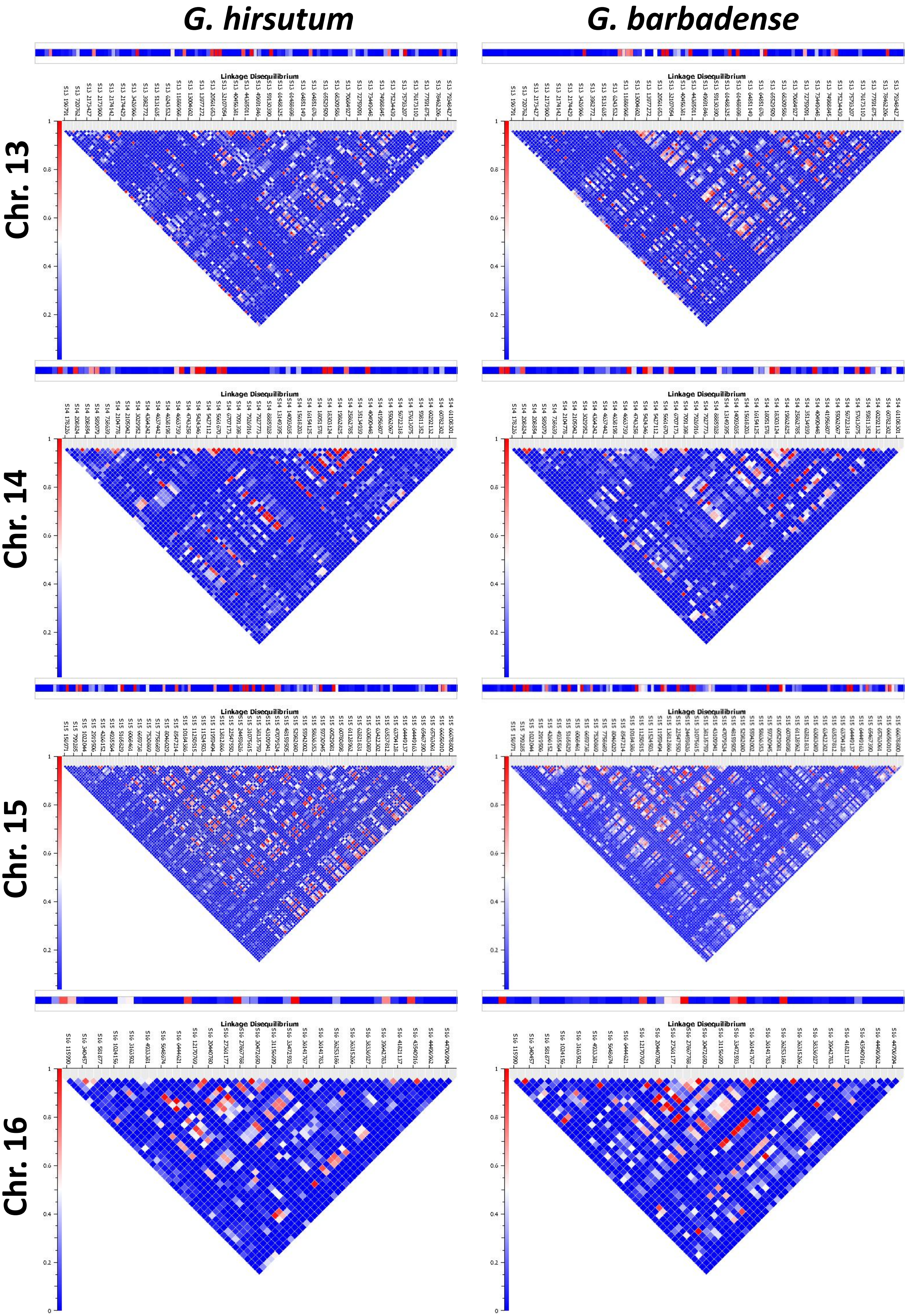
G. hirsutum
G. barbadense
Chr. 13
Chr. 14
Chr. 15
Chr. 16
Linkage Disequilibrium

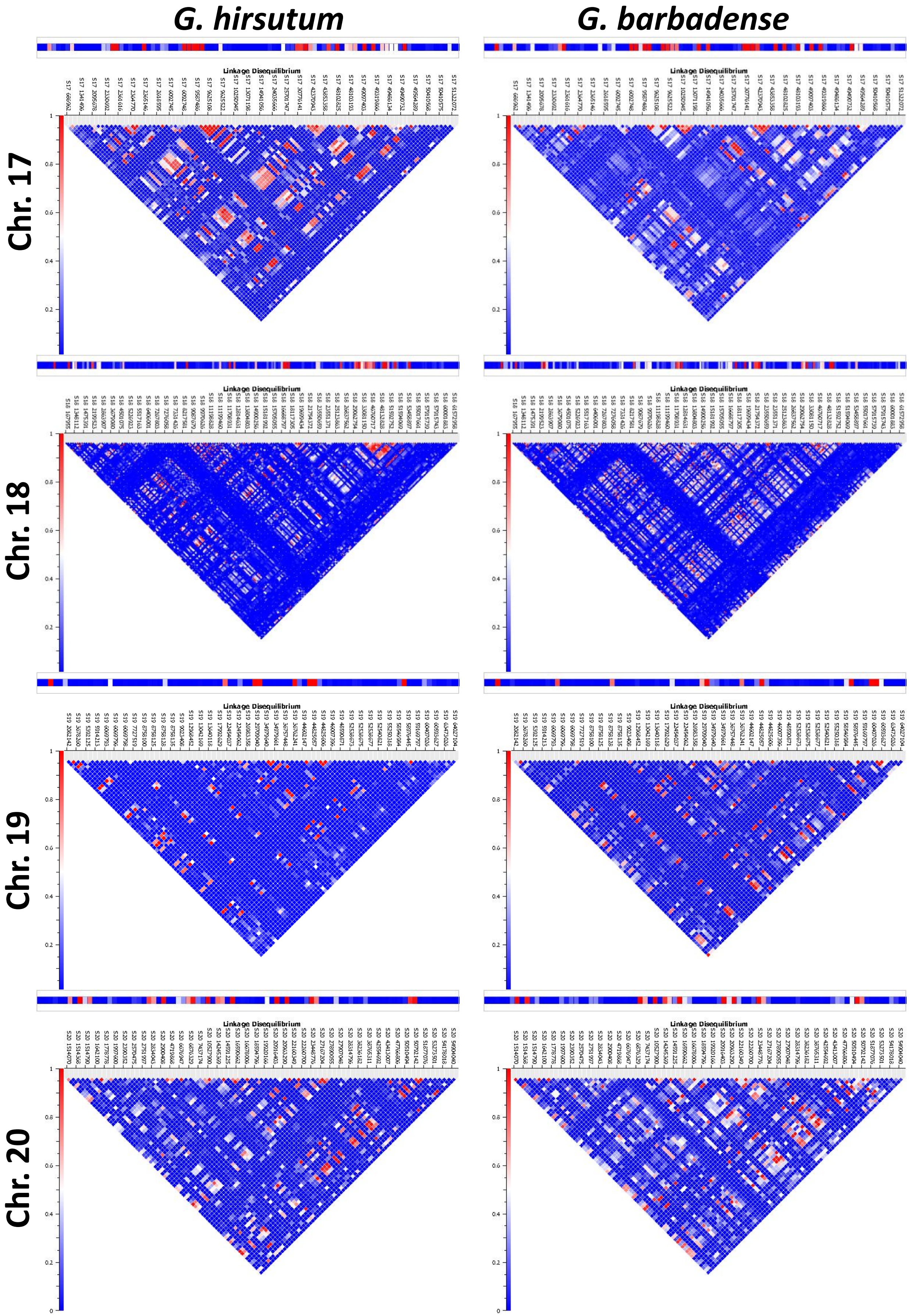
G. hirsutum
G. barbadense
Chr. 17
Chr. 18
Chr. 19
Chr. 20
Linkage Disequilibrium

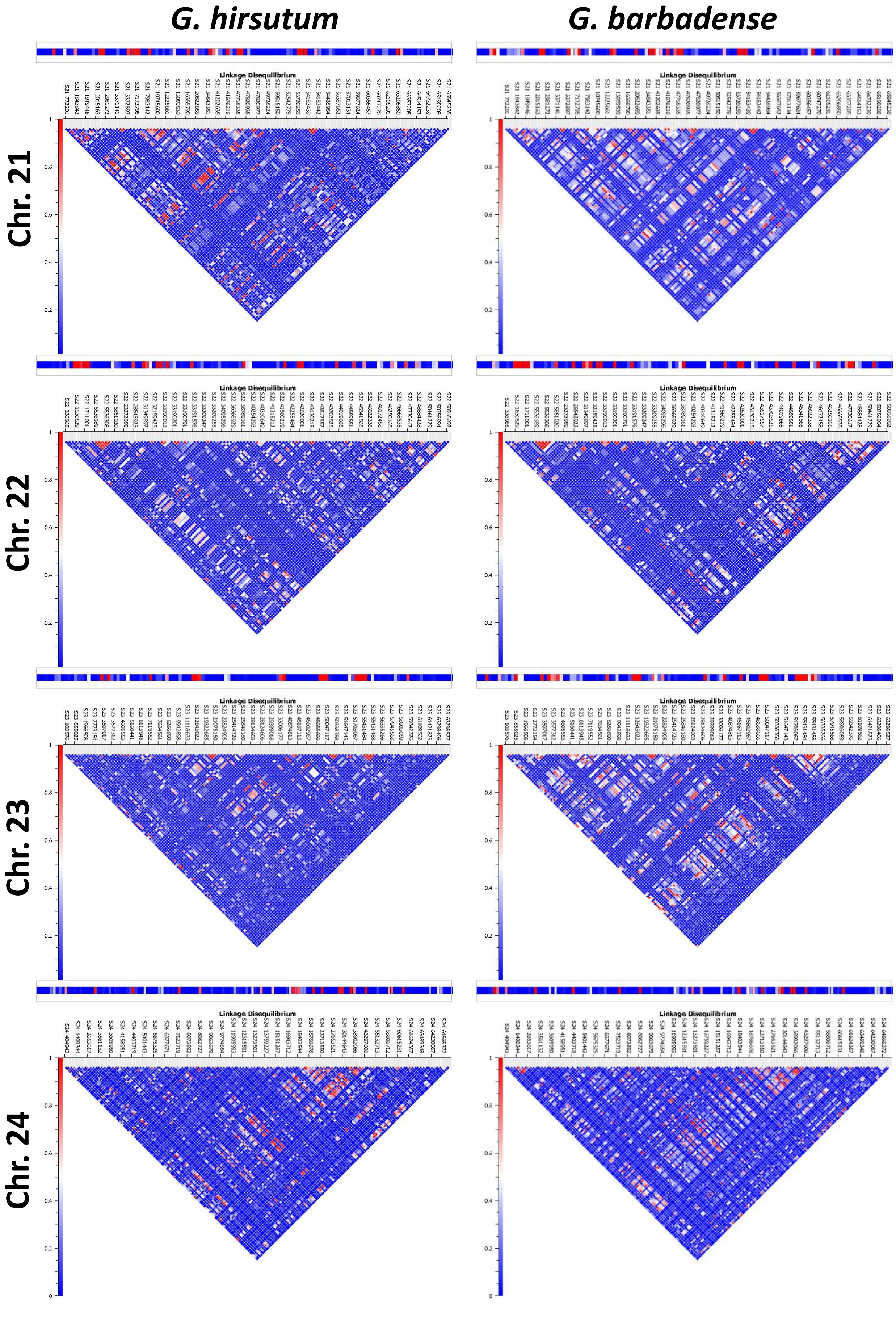
G. hirsutum
G. barbadense
Chr. 21
Chr. 22
Chr. 23
Chr. 24
Linkage Disequilibrium

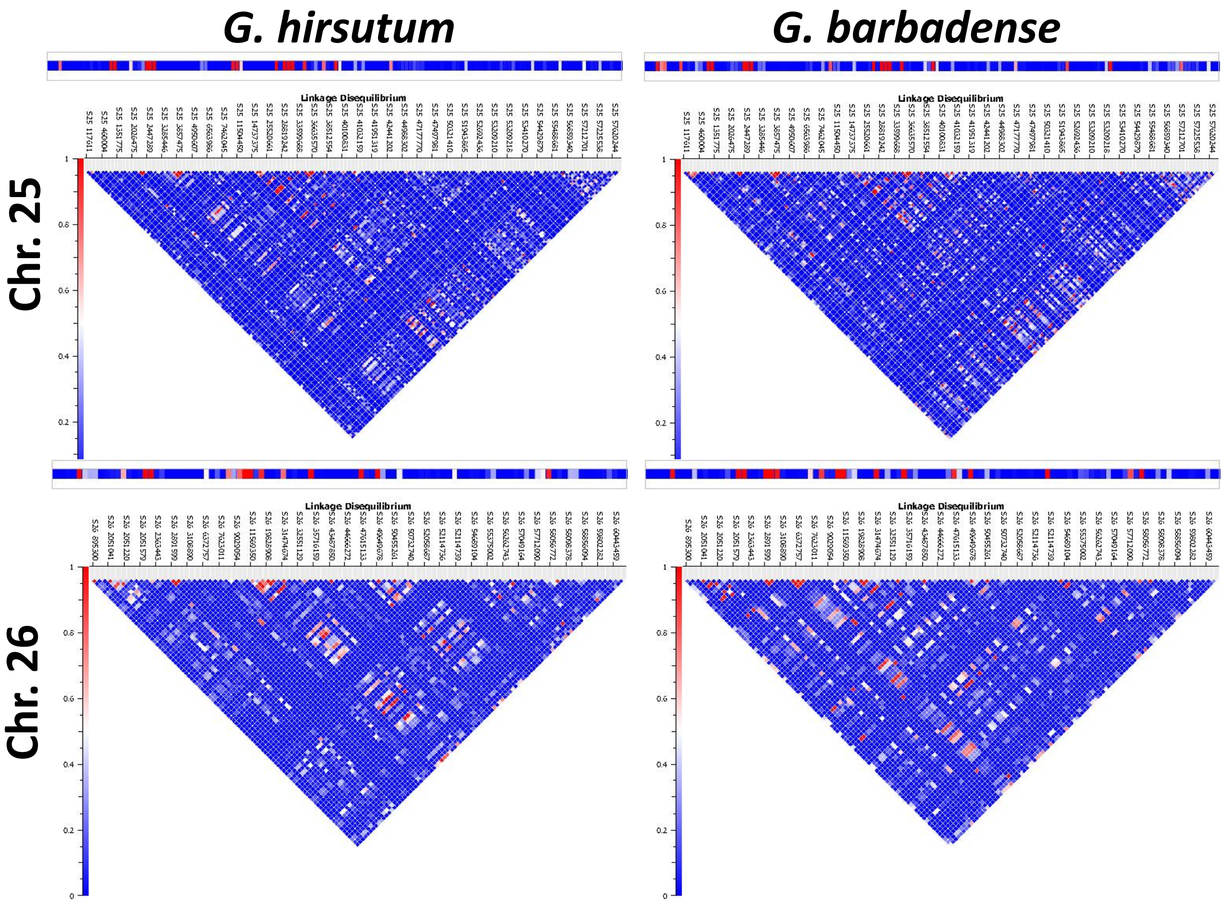

G. hirsutum
G. barbadense
Chr. 25
Chr. 26
Linkage Disequilibrium
